# Teriparatide and clodronate combination as a potential treatment for complex regional pain syndrome type I in delayed consolidation after foot surgery: a case report and review of the literature

**DOI:** 10.1186/s13256-024-04391-9

**Published:** 2024-03-08

**Authors:** F. Di Sacco, D. Antognetti, G. Ciapini, M. Nicastro, M. Scaglione, V. Bottai

**Affiliations:** 1 Department of Orthopedics and Trauma Surgery, University of Pisa, Pisa, Italy; 2 Section of Neurorehabilitation, Department of Translational Research and New Technologies in Medicine and Surgery, University of Pisa, Pisa, Italy; 3 Department of Anaesthesia and Critical Care Medicine, Azienda Ospedaliera Universitaria Pisana, University of Pisa, Pisa, Italy

**Keywords:** Complex regional pain syndrome type I, Clodronate, Teriparatide, Foot surgery

## Abstract

**Background:**

Complex regional pain syndrome type I is a pathological condition characterized by an exaggerated response of tissues to low or moderate pain stimuli. The exact pathogenesis and optimal medical treatment for complex regional pain syndrome type I are still not fully understood, although bisphosphonates have shown positive effects in reducing pain. Foot surgery can be complicated by the development of complex regional pain syndrome type I, leading to functional decline and difficulties in weight-bearing.

**Case presentation:**

The authors present a clinical case involving complex regional pain syndrome type I that developed after surgical foot arthrodesis. The patient, a 42-year-old Caucasian male, did not respond to clodronate treatment but experienced successful outcomes upon the addition of teriparatide, which effectively stimulated the healing of arthrodesis.

**Conclusion:**

Teriparatide cannot be considered the primary treatment for complex regional pain syndrome due to insufficient solid clinical data. However, when complex regional pain syndrome is associated with or caused by delayed union, teriparatide can be used to address the underlying cause of complex regional pain syndrome.

## Background

Complex regional pain syndrome (CRPS) is a challenging regional pain disorder characterized by persistent and often severe pain and accompanied by sensory, motor, and autonomic abnormalities.

It typically arises from low or moderate external stimuli, although the exact underlying mechanisms remain elusive. The development of complex regional pain syndrome type I (CRPS-I) involves a combination of factors, including dysregulation of the autonomic nervous system, neuropathic inflammation, genetic predisposition, and psychological influences. Together, these factors contribute to the onset of CRPS-I and highlight the multifaceted nature of this condition [1].

Molfetta *et al*. include CRPS-I within the context of bone marrow edema syndrome, with a specific focus on metabolic bone impairment [2].

The utilization of the Budapest criteria, such as disproportionate pain, swelling, skin discoloration, and sweating are characteristic signs, ensures a standardized approach to CRPS-I diagnosis, facilitating early intervention and appropriate management strategies [3, 4].

An interdisciplinary approach is recommended for the management of CRPS-I, and one of the pharmacological treatment options that has shown effectiveness in reducing pain is the use of bisphosphonates [5–7].

Teriparatide is a therapeutic agent that stimulates the formation of new bone tissue and helps improve the structure of bones affected by osteoporosis. Studies have shown that teriparatide promotes bone formation more than bone resorption, both biochemically and histologically. It works by directly stimulating bone formation in active remodeling sites (remodeling-based bone formation) and previously inactive bone surfaces (modeling-based bone formation), as well as by increasing the initiation of new remodeling sites [8].

The existing evidence substantiates that teriparatide has the potential to enhance callus formation and facilitate the healing of fractures in cases of delayed union and nonunion [9, 10].

The incidence of CRPS after foot surgery varies across studies, ranging from 0.5% to 7% [11]. Several risk factors have been identified, including prior history of CRPS, traumatic injury, complex surgeries, and prolonged immobilization. The exact mechanisms underlying the development of CRPS remain unclear, but it is thought to involve a combination of peripheral and central nervous system abnormalities.

## Case presentation

A 42-year-old Caucasian male was admitted at our clinic with a medical history of bilateral congenital clubfoot, which had been treated surgically through a right foot arthrodesis performed 13 years before. The patient presented with significant pain and instability in his left foot, resulting in a marked impairment of his ambulatory capacity. He indicated an allergic reaction to Cefixoral and denied any usage of tobacco or alcohol.

At clinical examination, the patient exhibited palpable pedis pulses and an intact neurological status. His walking pattern was characterized by an antalgic gait, and a 15° inward angulation of the forefoot was evident during weight-bearing. Additionally, a rigid inward angulation of the rearfoot was observed when the foot was in a neutral calcaneal stance. The sites of previous subtalar and midtarsal arthrodesis displayed rigid fixation, with elicitation of discomfort during attempts to achieve joint movement. Painful, thickened skin tissue was found diffusely on the undersurface of the base and head of the fifth metatarsal bone, accompanied by mild swelling.

**Fig. 1:**
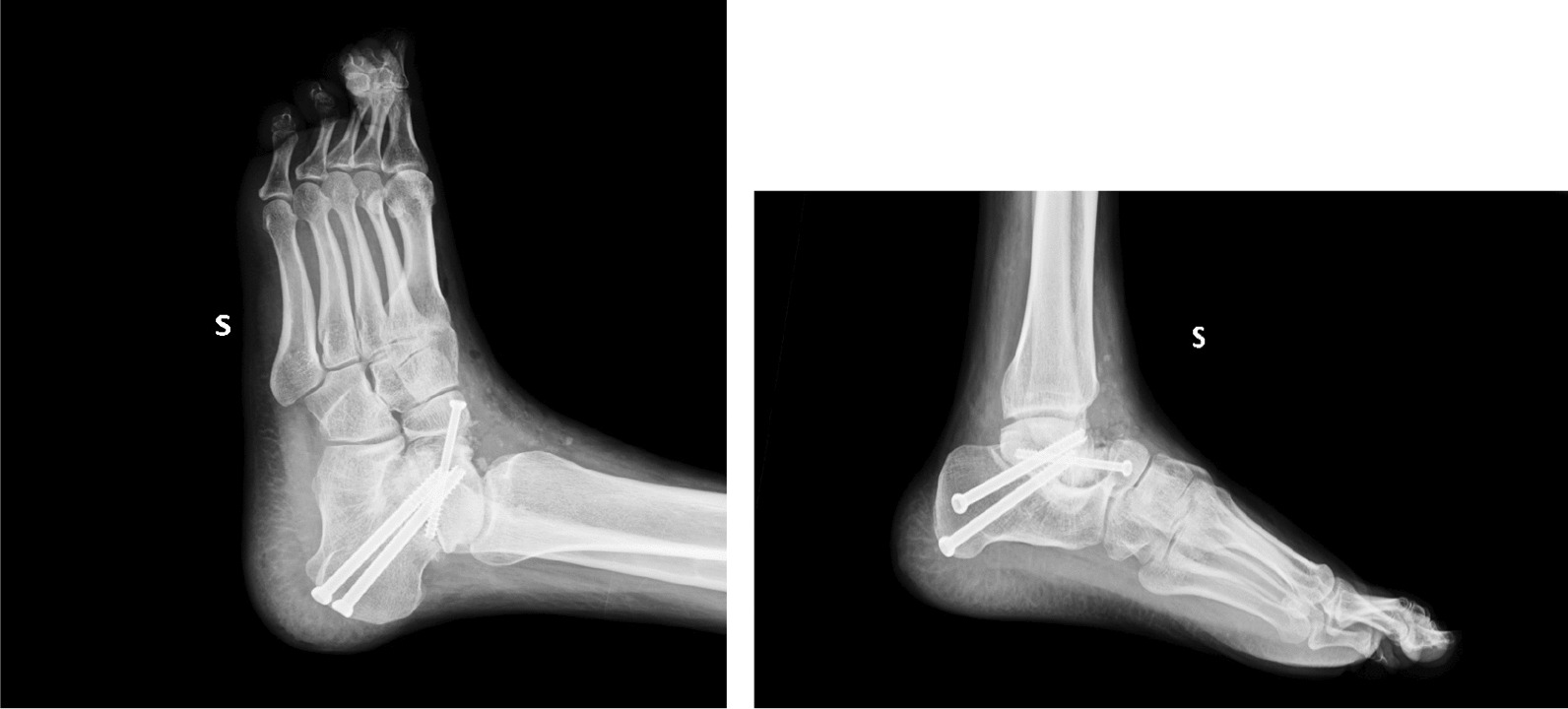
Post-operative X-Ray after surgery

Based on the clinical presentation and examination results, a diagnosis of misalignment involving both the rearfoot and the forefoot was established. Surgical intervention was performed to address this, involving subtalar arthrodesis stabilized with screws. The procedure was executed without any immediate surgical complications (Fig. 1)*.*

However, during the 6-month postoperative follow-up, the patient reported exacerbating pain around the external ankle area and noticeable swelling. These symptoms intensified during ambulation and did not respond to conservative or functional kinetic interventions.

**Fig. 2:**
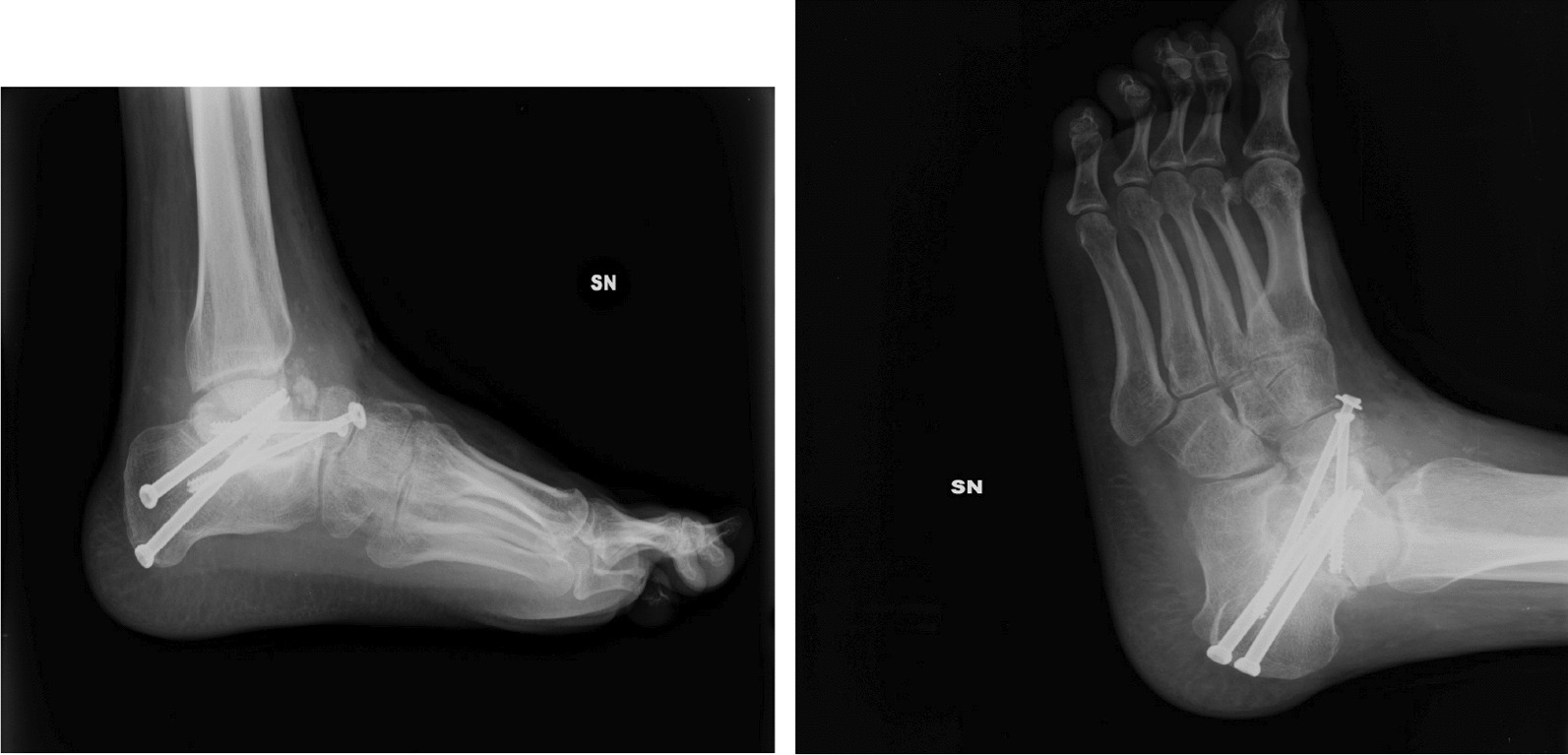
Post-operative x-ray of revision arthrodesis

Clinical evaluation indicated a 15° upward angulation of the forefoot and restricted range of motion in ankle joint flexion–extension and inversion–eversion, contributing to the patient’s distressing symptoms. Further investigation unveiled a failure of bone fusion at the subtalar joint level and evidence of reduced bone density in the foot and ankle region. To address these issues, a revision arthrodesis procedure was undertaken, during which bone marrow stem cells were harvested from the right iliac crest and employed to bridge the bone gap. The revision surgery also involved additional screw fixation at the arthrodesis site. (Fig. 2)*.*

Following the revision procedure, the patient’s foot was immobilized using a plaster cast and kept nonweight-bearing for a period of 2 months. Upon removal of the cast and initiation of weight-bearing activities after 3 months, the patient experienced discomfort upon loading the foot, accompanied by localized swelling, redness, and increased perspiration of the skin.

**Fig. 3:**
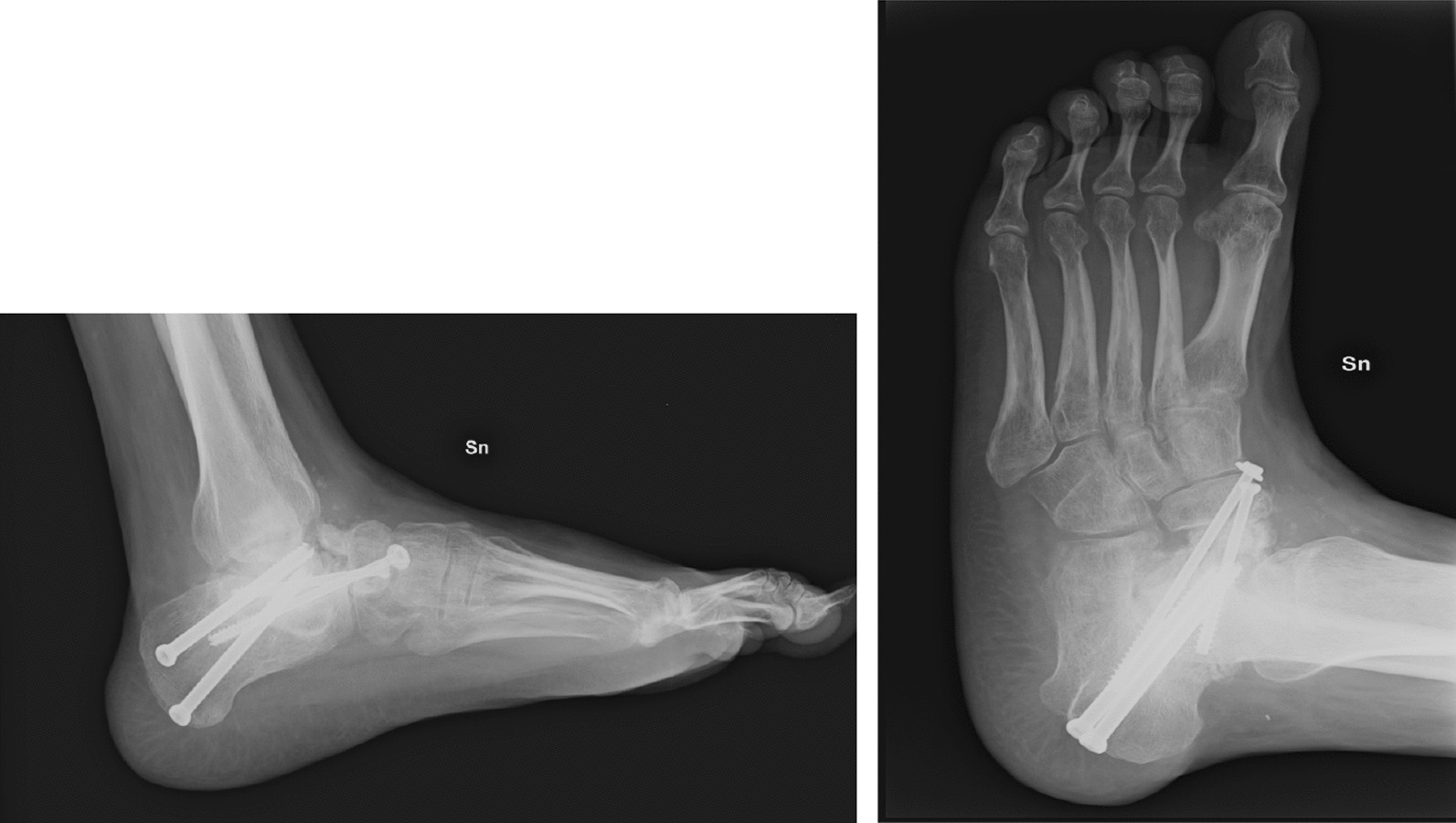
4 months post-operative x-ray

In response to these symptoms, the patient was prescribed clodronate, intramuscularly, once every 14 days and 6–8 hours a day of magnetotherapy for 21 days. Radiographic imaging acquired 4 months postrevision surgery revealed incomplete fusion of the arthrodesis site and signs of irregular bone density in the region. (Fig. 3)*.*

**Fig. 4:**
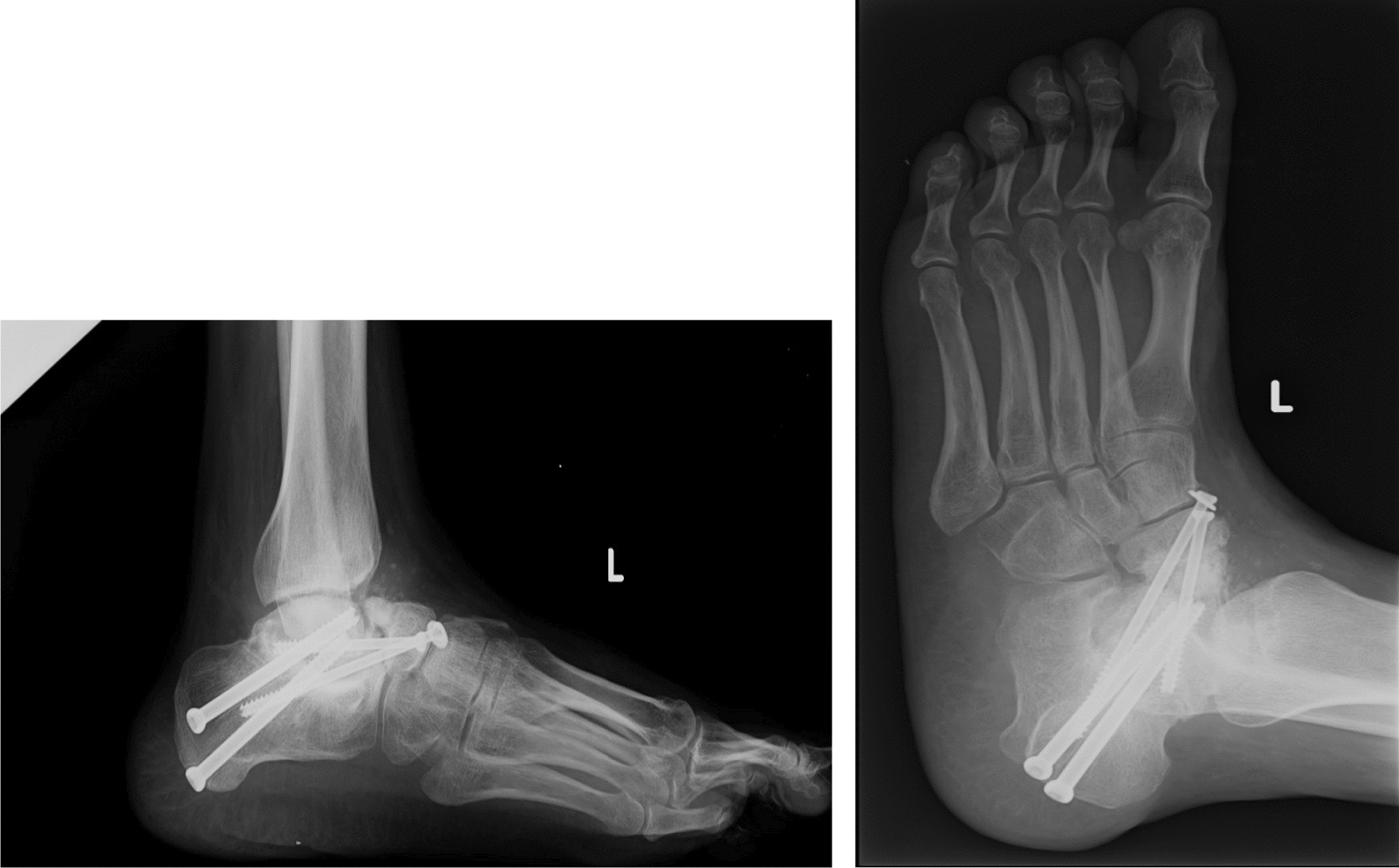
3 months x-ray after therapy

Subsequently, to promote arthrodesis, fusion was introduced with subcutaneous teriparatide 20 mcg/die, in association with clodronate and oral supplementation of calcium and vitamin D. Within a month of starting teriparatide therapy, the patient displayed both clinical and radiographic improvement. By the 3-month mark, a complete resolution of the clinical and radiographic manifestations was achieved. (Fig. 4)*.*

Patient could walk without aids and came back to his normal activity as before the surgery.

## Discussion

The incidence of complex regional pain syndrome type 1 (CRPS-I) is higher in females than males, and it is most commonly seen in middle-aged individuals. CRPS-I can occur as a result of trauma or surgery, typically manifesting 4–6 weeks after the initial cause. Postsurgical CRPS incidence varies, but it has been reported to be more prevalent in foot surgeries. The incidence of CRPS after foot surgery varies across studies, ranging from 0.5% to 7% [1, 11, 12].

The pathogenesis of postsurgical CRPS-I is not fully understood, but it is believed to involve abnormal inflammatory and immune responses. Surgical procedures on the foot pose specific risk factors for CRPS development, including the manipulation of peripheral nerves, bone and tissue trauma, and immobilization periods. These factors may contribute to nerve dysfunction and the subsequent development of CRPS.

CRPS-I typically progresses through several clinical phases. These include an acute inflammatory stage characterized by pain, swelling, and changes in skin temperature and color. This may be followed by a dystrophic stage where the affected limb experiences changes in hair growth, skin texture, and nail appearance. In some cases, CRPS-I may progress to a chronic stage, resulting in persistent pain and functional impairment [1].

Bisphosphonates, such as clodronate, have been investigated for their potential utility in treating CRPS. These medications are thought to inhibit bone resorption and modulate inflammatory responses. However, their use in CRPS remains a topic of debate, and their effectiveness is not yet fully established [5].

In this specific case CRPS developed 2 months after cast removal and weight-bearing initiation; previous treatments with magnets and clodronate did not yield a satisfactory response. In this situation, the rationale for the use of teriparatide was based on identifying delayed healing of the arthrodesis as the cause of CRPS-I. Teriparatide is known to promote bone formation and healing. Although there is limited literature on the use of teriparatide for CRPS [13, 14], the patient showed resolution of symptoms within 6 weeks, coinciding with positive healing progression.

It is worth noting that previous treatments with magnets and clodronate had been attempted, but significant clinical improvement only occurred after the introduction of teriparatide after a few weeks.

It is important to remember that the information provided here is a general overview and should not replace medical advice. The use of teriparatide or any other medications for CRPS should be discussed with a healthcare professional, considering the individual patient's specific circumstances and medical history.

## Conclusion

Teriparatide cannot be considered the treatment of choice for complex regional pain syndrome (CRPS) due to a lack of robust clinical data supporting its efficacy. However, if CRPS is associated and/or caused by delayed fracture healing, teriparatide may be utilized to address the underlying cause of CRPS.

## Data Availability

Not applicable.
